# Age-Dependent Sex Difference of the Incidence and Mortality of Status Epilepticus: A Twelve Year Nationwide Population-Based Cohort Study in Taiwan

**DOI:** 10.1371/journal.pone.0122350

**Published:** 2015-03-31

**Authors:** Cheung-Ter Ong, Shew-Meei Sheu, Ching-Fang Tsai, Yi-Sin Wong, Solomon Chih-Cheng Chen

**Affiliations:** 1 Department of Neurology, Ditmanson Medical Foundation Chia-Yi Christian Hospital, Chiayi city, Taiwan; 2 Department of Nursing, Chung Jen Junior College of Nursing, Health Science and Management, Chia-Yi, Taiwan; 3 Department of Medical Research, Ditmanson Medical Foundation Chia-Yi Christian Hospital, Chiayi city, Taiwan; 4 Department of Family Medicine, Ditmanson Medical Foundation Chia-Yi Christian Hospital, Chiayi city, Taiwan; 5 Department of Pediatrics, School of Medicine, Taipei Medical University, Taipei, Taiwan; University of Modena and Reggio Emilia, ITALY

## Abstract

Status epilepticus (SE) is a serious neurologic emergency associated with a significant mortality. The objective of this study is to investigate its epidemiology in terms of age- and sex-specific incidences and mortality. By using the Taiwan National Health Insurance Research Database during 2000 to 2011, we identified hospitalized patients with a discharged diagnosis of SE and calculated the incidence and in-hospital mortality of SE with respect to age and sex. The overall incidence of SE was 4.61 per 100,000 person-years, which displayed a “J-shaped” distribution by age with a little higher under the age of 5 and highest over 60 years. The male-to-female rate ratio was 1.57 and it demonstrated a “mountain-shape” across ages with the peak at 45 to 49 years old. The in-hospital mortality was significantly lower in males (7.38%) than in females (11.12%) with an odds ratio of 0.64 (95% CI 0.56-0.72). Notably, the in-hospital mortality for females increased rapidly after the age of 40 to 45 years. The multivariate analysis found males had a significantly lower risk of mortality than females after, but not before, 45 years of age with an odds ratio of 0.56 (95% CI 0.49-0.65). Sex and age are crucial factors associated with the incidence and in-hospital mortality of SE. The females over 45 years of age have a higher risk of occurrence and mortality from SE. The underlying mechanism deserves further study.

## Introduction

Status epilepticus (SE) is a serious neurologic emergency and is often associated with significant mortality [1]. Approximately 20% of patients with SE have been shown to die within the first 30 days [2,3], and patients who survive often develop neurologic complications such as encephalopathy (6% to 15%) and focal neurologic deficits (9% to 11%) [4,5]. The incidence of SE in America and Europe were between 6.2 and 41 per 100,000 person-years, and case fatality ranged from 7% to 39%, respectively [6–14]. Though there were some epidemiological studies examining SE [6–14], most of them were in Western countries, and very few studies were from Asian countries [15,16]. Some studies found males are more likely to suffer from SE than females [7–9]. However, the sex differences in SE occurrence and its mortality by age have not yet been well studied.

Because older patients are at higher risk of SE [7,9] and populations are visibly aging, the occurrence of SE will subsequently increase and become an important issue. Therefore, by using the data from Taiwan National Health Insurance Research Dataset (NHIRD), we performed a population-based cohort study to analyze the incidence and mortality of all-hospital admissions for SE specifically examining the effect of age and sex from 2000 to 2011.

## Materials and Methods

### Data source

This is a population-based study using the data obtained from the Taiwan NHIRD, which is a claim database and has been extensively used for many studies [17–19]. In Taiwan, the National Health Insurance (NHI) program has been initiated since 1995, which is compulsory to all Taiwanese citizens except criminals and military personnel and cover over 99% (about 23 million) of the population [20]. Medical institutions received reimbursement from NHI by providing comprehensive medical services including almost all outpatient and inpatient cares. Data in this study were retrieved from admission claims of NHIRD from 1997 to 2011. All of the individual’s admission records during this period can be linked by using encrypted identification number. In each admission record, up to five discharge diagnoses were available and coded in International Classification of Diseases, Ninth Revision, Clinical Modification (ICD-9-CM). This study have been reviewed and approved by the Institutional Review Board of the Ditmanson Medical Foundation Chia-Yi Christian Hospital, Taiwan.

### Study subjects and definition

An ICD-9-CM code of 345.3 denoted in any of the first three diagnoses codes was used to identify an episode of SE. SE was defined as continuous seizure for at least 5 minutes and/or repeated seizure without returning to baseline and/or the patient’s electroencephalography (EEG) consistent with SE [14]. Patients who had ever hospitalized due to SE from 2000 to 2011 were included. Only those who had no SE-related hospitalizations during a wash-out period from 1997 to 1999 were considered as the incident cases. Despite patients may experience several attacks of SE during the study period, we only included the first hospitalization into our analysis. Patient’s characteristics (i.e. age and sex), calendar year of hospitalization, and in–hospital mortality potentially related to SE were studied. Age at the first diagnosis was categorized into five groups: 0 to 4, 5 to 19, 20 to 44, 45 to 59 and over 60 years old. To investigate the temporal trend of incidences, we separated the study period into four sub-periods and analyzed the patients’ characteristics every three years.

### Statistical analysis

We calculated the incidence rate of SE per 100,000 person-years and further stratified by sex, age groups, and sub-periods of calendar year from 2000–2011. The numerator of the incidence rate was the number of incident cases, and the denominator was the total population in that year, which was obtained from the Department of Statistics in the Ministry of the Interior of Executive Yuan in Taiwan. The temporal trend of incidences was tested by Poisson regression analysis. Rate ratios and 95% confidence intervals (95% CI) were estimated under normal assumptions of the Poisson distribution. Male-to-female rate ratios and age-specific incidences for both males and females were plotted to depict the age and sex effects on the incidences of SE. We also reported in-hospital mortality across age groups. Crude and adjusted odds ratios (ORs) with 95% CI were estimated for associated factors of in-hospital mortality such as sex, age, and sub-periods of calendar year by using univariate and multivariate logistic regression models. Data analysis was performed by using SPSS software, Version 21 of the SPSS System for Windows (version 21.0; IBM Corporation, Somers, NY, USA). A two-tailed P-value < 0.05 was considered to be statistically significant.

## Results

### Incidence

**Fig 1:**
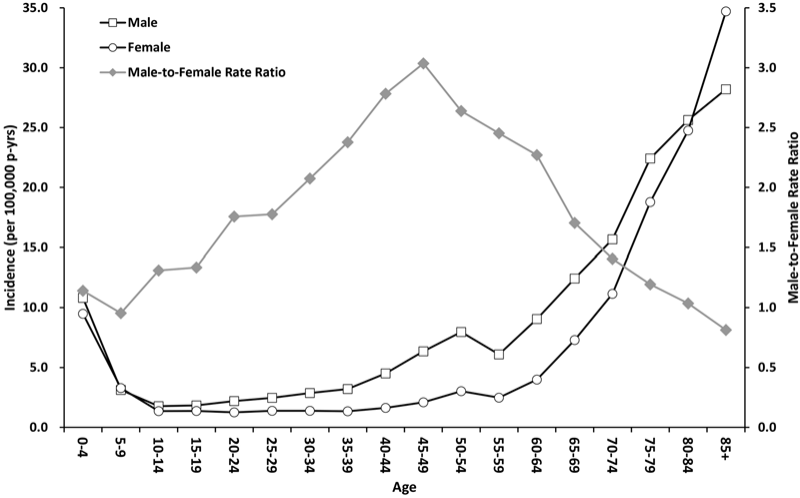
Incidence and male-to-female risk ratios of status epilepticus hospitalizations by age in Taiwan from 2000–2011.

From 2000 to 2011, a total of 12,627 patients were identified with a first hospitalization for SE with an overall incidence of 4.61 per 100,000 person-years during the study period (Table 1). Age stratification revealed a “J-shaped” distribution with a higher incidence at under 5 years of age (10.18/100,000 person-years) and over 60 years of age (approaching 13.85/100,000 person-years, Fig 1). The incidences showed an increasing trend from 3.87 to 5.08 per 100,000 person-years over the 12-years study period (Table 1). When further categorizing by age groups, the increasing trend was significant in patient age groups over 20 years of age (Table 2).

**Table 1:** Incidence of SE hospitalizations in Taiwan from 2000–2011.

Parameter	No. of case	%	Incidence Rate (per 100,000 person-years)	Relative risk	95% CI^a^
All patients	12627	100.00	4.61	-	-
Sex					
Female	4837	38.31	3.58	Reference	-
Male	7790	61.69	5.62	1.57	1.51–1.63
Age, y					
0–4	1431	11.33	10.18	Reference	-
5–19	1263	10.00	2.26	0.22	0.21–0.24
20–44	2493	19.74	2.22	0.22	0.20–0.23
45–59	2295	18.18	4.22	0.42	0.39–0.44
≥ 60	5145	40.75	13.85	1.36	1.28–1.44
Year					
2000–2002	2602	20.61	3.87	Reference	-
2003–2005	3178	25.17	4.67	1.21	1.15–1.27
2006–2008	3315	26.25	4.81	1.24	1.18–1.31
2009–2011	3532	27.97	5.08	1.31	1.25–1.38

SE indicates status epilepticus.

^a^CI, confidence interval.

**Table 2:** Trend of the incidences of patients with SE from 2000–2011 by both sexes and age groups.

Incidence (per 100,000 person-years)	2000–2002	2003–2005	2006–2008	2009–2011	P value for trend
Sex					
Female	3.11	3.61	3.68	3.91	<0.001
Male	4.60	5.70	5.92	6.24	<0.001
Age, y					
0–4	8.65	11.84	10.47	9.99	0.1333
5–19	2.05	2.36	2.45	2.20	0.2889
20–44	1.93	2.40	2.23	2.33	0.0090
45–59	3.83	4.01	4.36	4.56	0.0014
≥60	11.45	13.65	14.72	15.12	<0.001

SE indicates status epilepticus.

### Sex ratio of incidence

The incidence of SE was higher in males than females with an overall male-to-female rate ratio of 1.57. (95% CI 1.51–1.63, in Table 1) The male-to-female rate ratio changed by age and showed a “mountain-shape” with the peak at the age group of 45 to 49 years in Fig 1.

### In-hospital mortality and its sex ratio

**Fig 2:**
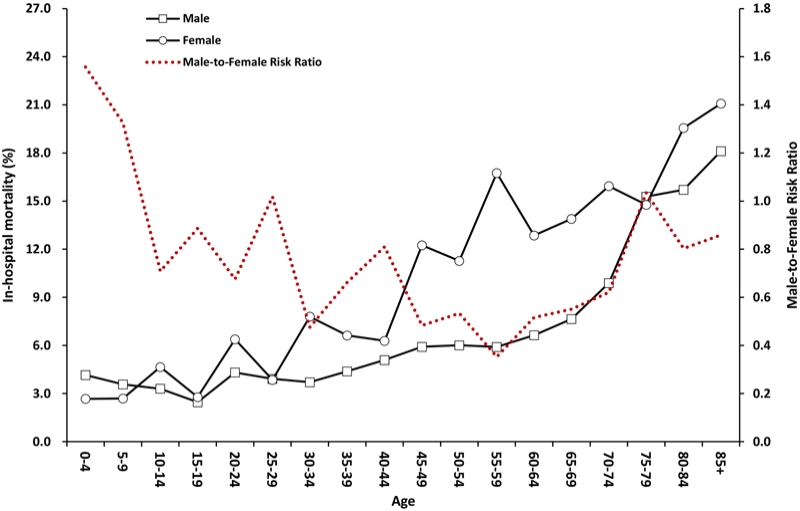
In-hospital mortality and male-to-female risk ratios of status epilepticus hospitalizations by age in Taiwan from 2000–2011.

The overall in-hospital mortality was 8.81%, significantly lower in males than in females (7.38% vs. 11.12% in Table 3, OR = 0.64, 95% CI 0.56 to 0.72). The mortality rate was the lowest (3.17%) at ages of 5 to 19 years and approached 13.96% at over 60 years of age. Compared to 2000 to 2002, the in-hospital mortality was significantly lower in later years. (Table 3) When stratified by age, the male-to-female risk ratio showed an “U-shaped” distribution, and most of the ratios were less than one. Stratified by sex, the mortality showed a rapid increase for female patients at 40 to 45 years of age, but an obvious increase for male patients did not occur until over 70 years of age. (Fig 2) Compared to females, the in-hospital mortality was significantly lower for males over 45 years of age (OR = 0.56, 95% CI 0.49 to 0.65).

**Table 3:** Multivariable logistic regression analysis of in-hospital mortality for patients with SE.

Parameter	Case fatality,%	Univariate		Multivariate	
		OR^a^	95% CI^b^	OR^a^	95% CI^b^
All patients	8.81	-	-	-	-
Sex					
Female	11.12	Reference	-	Reference	-
Male	7.38	0.64	0.56–0.72	0.66	0.58–0.75
Age, y					
0–4	3.49	Reference	-	Reference	-
5–19	3.17	0.90	0.59–1.38	0.91	0.59–1.38
20–44	4.93	1.43	1.03–2.01	1.54	1.10–2.16
45–59	7.93	2.38	1.73–3.28	2.64	1.92–3.65
≥60	13.96	4.48	3.34–6.00	4.65	3.46–6.24
Year					
2000–2002	10.22	Reference	-	Reference	-
2003–2005	8.09	0.77	0.64–0.92	0.75	0.62–0.90
2006–2008	8.36	0.80	0.67–0.96	0.73	0.61–0.87
2009–2011	8.86	0.85	0.72–1.01	0.74	0.62–0.88

SE indicates status epilepticus;

^a^OR, odds ratio;

^b^CI, confidence interval.

The odd ratio of in-hospital mortality adjusted for sex, age and year using logistic repression.

## Discussion

This is a nationwide population-based cohort study in Taiwan. We found that the incidence of SE displayed a “J-shaped” distribution by age, with a higher incidence at under age 5 and over 60 years of age. The male-to-female rate ratio of SE cases was dynamic and demonstrated a “mountain-shape” by age with the highest peak at 45 to 49 years of age. The in-hospital mortality of SE also increased rapidly in females over 45 years of age and in males over 70 years of age. The large-scale epidemiological evidence in Taiwan could provide very detailed age and sex effect on the occurrence and mortality of SE.

The overall incidence of SE in Taiwan was 4.61 per 100,000 person-years, which is similar to the 5.10 per 100,000 person-years reported in Thailand [16]. However, the incidence was much lower than the 6.2 to 41 per 100,000 person-years reported in American and European countries [6–14]. Wu et al., has demonstrated that Asian people had a lower incidence of SE than whites and blacks in California, USA [10]. Thus, we thought the discrepancy of incidences could be due to ethnic difference. Moreover, one possible reason is the data collection. Both Thailand and Taiwan have national health insurance system and the studied data were population-based, which is different from the institutes-based data used in European and US studies. Additionally, the “J-shaped” distribution by age in the incidence of SE in our study was similar to a previous study [10,14], and the incidence was much higher for older patients [7–14]. Given the aging population, SE may become an increasingly important health issue in the future. Our results also found that more males have SE than females (Tables 1 and 2), which agrees with previous studies [7–9,14,16]. Moreover, we found that male-to-female rate ratio of SE occurrence changed by age and displayed a “mountain-shape” with a peak the ages of 45 to 49 years of age corresponding to the usual period of female menopause (Fig 1). According to the study of Wu et al. [10], we have grouped the potential etiologies or comorbidities of SE patients into 15 categories in the S1 Table. Late effect of stroke or brain injury and stroke were two major etiologies of patients with SE in Taiwan, compatible with previous studies [9,10,21].

The sex effect on mortality of SE is still controversial. One study in USA and the other in Thailand suggested a slightly higher mortality rate in males than in females [14,16]. But another study in California found the mortality rates between two sexes were not different [10]. In this study, we found that males have a significantly lower risk of mortality than females (OR = 0.64, 95% CI 0.56–0.72 in Table 3), which corroborates with the results of National Inpatient Sample databases in the United States [21]. We thought such discrepancy may come from several reasons, like the age distribution of study population, the frequency of underlying etiologies and complications of SE [10,14,16,21].

In Table 3, the univariate model showed a remarkable reduction of in-hospital mortality after 2002, which was followed by a gradual increase from 2003 to 2011. This change may be due to the increasing proportion of elderly patients in Taiwan. However, after adjusting the age and sex effect in the multivariate model, in-hospital mortality was consistently lowered from 2000 to 2011. We considered the continuous decrease of in-hospitality mortality of SE may be related to general improvements in the health care system and early treatment for SE patients in Taiwan.

Notably, the mortality increases were different in both sexes. In Fig 2, there was a jump of in-hospital mortality for female patients after the age group of 45 to 49 years of age, but the mortality for male patients increased slowly and finally reached a peak after 70 years of age. We speculate the increase of in-mortality for male patients by age was due to the aging effect of population. But such rapid increase of in-hospital mortality for female patients should have other reasons. Previous studies suggested that death after the occurrence of SE is usually due to the underlying cause [2,22]. Our preliminary analysis found that anoxia and brain tumor were two major factors associated with 48.9% of mortality (S1 Table). However, the occurrences of these two etiologies did not show any sex or age relation pattern. Because 45 to 49 years of age is the usual period of female menopause, the dramatic increase of mortality could be related to the drop in the reproductive hormones during and after the menopause. Female reproductive hormones are known to have opposing effects on neuronal excitability [23] and decrease the seizure frequency of catamenial type of epilepsy [24,25]. Hormonal replacement therapy has been suggested in postmenopausal women with epilepsy to improve their seizure control as well as for better quality of life [26,27]. Thus, the protective effect of reproductive hormone may decline, and the occurrence and the in-hospital mortality of female patients with SE subsequently increase after the menopause.

### Limitations

Our data were retrieved from the Taiwan NHIRD which lacks some detailed clinical information such as the seizure type and how the patients were diagnosed. Some patients who had partial seizures secondary to generalized convulsions may also be diagnosed as SE. However, the coverage of NHI program in Taiwan is over 99% of all citizens and the accessibility to medical resources is quite good, almost every citizen in Taiwan can reach hospitals within one hour. Moreover, since SE is a life-threatening disease and all patients with SE would be sent to Emergency Department and then hospitalized for further care. Thus, we have confidence in the completeness of recruiting all SE cases in this nationwide dataset. The large number of cases over a long period of 12 years still can give us a representative picture of the sex differences of incidence and mortality of SE.

## Conclusions

Sex and age are critical factors involved in the incidence and in-hospital mortality of SE. The females over 45 years of age have a higher risk of the occurrence and mortality of SE. The underlying mechanism deserves further study.

## Supporting Information

S1 Table The comorbidities or potential etiologies of status epilepticus in 12,627 patients. (DOC)
