# Correction: Age-Dependent Sex Difference of the Incidence and Mortality of Status Epilepticus: A Twelve Year Nationwide Population-Based Cohort Study in Taiwan

**DOI:** 10.1371/journal.pone.0173841

**Published:** 2017-03-07

**Authors:** Cheung-Ter Ong, Shew-Meei Sheu, Ching-Fang Tsai, Yi-Sin Wong, Solomon Chih-Cheng Chen

There is an error in the fifth author’s affiliation. The correct affiliation is: Department of Pediatrics, School of Medicine, College of Medicine, Taipei Medical University, Taipei City, Taiwan.
