# Supplementary material for: Age-Dependent Sex Difference of the Incidence and Mortality of Status Epilepticus: A Twelve Year Nationwide Population-Based Cohort Study in Taiwan
Source: PLoS One. 2015 Mar 31;10(3):e0122350. doi: 10.1371/journal.pone.0122350 (PMC4380468; doi:10.1371/journal.pone.0122350)
Supplement: S1 Table — (DOC) [file pone.0122350.s001.doc]

**S1 Table. The comorbidities or potential etiologies of status epilepticus in 12,627** patients

| **Comorbidity** | **No.** | **%** | **No. of death** | **Mortality, %** |
| --- | --- | --- | --- | --- |
| Late effect of stroke or brain injury | 2026 | **16.04** | 78 | 3.85 |
| Stroke | 1148 | **9.09** | 160 | 13.94 |
| Central Nervous System infection | 559 | 4.43 | 85 | 15.21 |
| Cerebral palsy | 468 | 3.71 | 8 | 1.71 |
| Sodium imbalance | 460 | 3.64 | 36 | 7.83 |
| Anoxia | 439 | 3.48 | 107 | 24.37 |
| Developmental delay | 435 | 3.44 | 6 | 1.38 |
| Alcoholism | 394 | 3.12 | 10 | 2.54 |
| Hydrocephalus | 257 | 2.04 | 16 | 6.23 |
| Fever | 216 | 1.71 | 17 | 7.87 |
| Brain tumor | 192 | 1.52 | 47 | 24.48 |
| Central Nervous System anomalies | 103 | 0.82 | 4 | 3.88 |
| Subdural hematoma | 86 | 0.68 | 15 | 17.44 |
| Multiple sclerosis | 5 | 0.04 | 0 | 0 |
| Noncompliance | 2 | 0.02 | 0 | 0 |
